# Taurine ameliorates cellular senescence associated with an increased hydrogen sulfide and a decreased hepatokine, IGFBP-1, in CCl4-induced hepatotoxicity in mice

**DOI:** 10.1016/j.redox.2025.103640

**Published:** 2025-04-18

**Authors:** Akihiro Tsuboi, Hamida Khanom, Riki Kawabata, Takanori Matsui, Shigeru Murakami, Takashi Ito

**Keywords:** Taurine, Senescence, Hepatokines, Hydrogen sulfide, Liver injury, IGFBP-1

## Abstract

This study investigated the protective effects of taurine against cellular senescence and hepatokine secretion in a mouse model of carbon tetrachloride (CCl4)-induced chronic liver injury. Oral taurine administration by tap water containing 3 % taurine significantly attenuated liver damage, as evidenced by reduced serum AST, ALT level and hepatic lipid peroxidation. Importantly, hepatic taurine level is reduced in CCl4-induced injury model, while taurine administration recovered it. Moreover, taurine administration decreased the numbers of p21-positive senescent cells in liver tissue of CCl4-treated mice. Taurine increases hydrogen sulfide (H_2_S) in liver of normal mice, suggesting anti-oxidative role through H_2_S production by taurine. Furthermore, inhibition of CTH, which is an enzyme responsible for H_2_S production from cysteine, by propagylglycine attenuated malondialdehyde-lowering effect of taurine in liver of CCl4-treated mice. Moreover, we found taurine treatment lowers insulin-like growth factor binding protein-1 (IGFBP-1) in liver of normal mice. Importantly, while chronic CCl4 injection caused an induction of IGFBP-1, taurine administration blocked it. These findings suggest that taurine exerts its protective effects by attenuating cellular senescence, which is associated with enhancing H_2_S production and inhibiting IGFBP-1 expression. This study highlights the potential of taurine as a therapeutic strategy for mitigating chronic liver injury by producing H_2_S and targeting IGFBP1.

## Highlights

• Taurine reduces p21-positive senescent cells, alleviating oxidative damage in CCl4-induced chronic liver injury.
• Taurine enhances hepatic hydrogen sulfide production, mediating its antioxidant defense and anti-senescence properties.
• Inhibiting H_2_S synthesis attenuates taurine's protective effect, confirming its pivotal role in redox homeostasis.
• Taurine inhibits CCl4-induced hepatokine IGFBP-1 expression.
• Our findings suggest a novel taurine–H_2_S/IGFBP-1 axis with implications for chronic liver disease and senescence research.

## Graphical abstract

**Figure:**
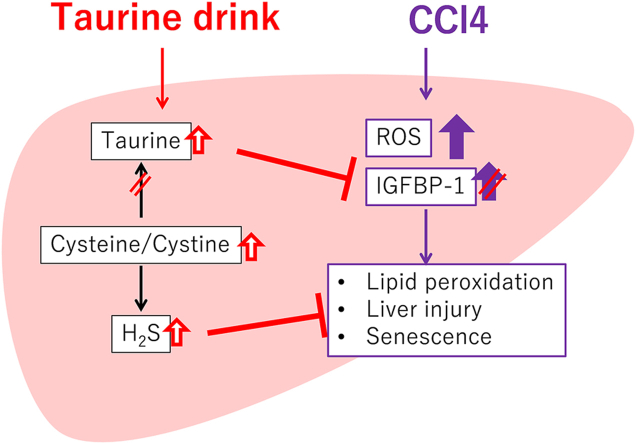

## Abbreviations

ALT: alanine aminotransferase
AST: aspartate aminotransferase
CCl_4_: carbon tetrachloride
CSAD: cysteine sulfinic acid decarboxylase
CTH: cystathionine γ-lyase
GSH: glutathione
H_2_S: hydrogen sulfide
IGFBP-1: insulin-like growth factor binding protein-1
MDA: malondialdehyde
PPG: propargylglycine
SASP: senescence-associated secretory phenotype
TGF-β: traforming growth factor-β
TNF-α: tumor necrosis factor-α

## 1 Introduction

Cellular senescence is a permanent cessation of cell division, in which some cells do not die but survive and accumulate, affecting surrounding cells [1]. Accumulated senescent cells secrete cytokines and growth factors, which induce chronic inflammation and aging of surrounding cells, causing age-related systemic functional decline. Cellular senescence is not only caused by aging, but also by DNA damage due to oxidative stress in pathological stresses and diseases. In the liver, its involvement in chronic liver diseases such as cirrhosis has been suggested [[2], [3], [4]]. Senescent cell has been found in various diseases such as alcoholic liver disease, non-alcoholic steatohepatitis, and viral hepatitis. Cellular senescence contributes to development of metabolic dysfunction and fibrosis in liver. Therefore, it may be possible to inhibit the progression of liver diseases by controlling cellular senescence. In addition, since hepatocyte senescence induces secretion factor-mediated senescence and dysfunction of multiple organs [5], control of hepatocyte senescence is beneficial for the prevention and treatment of various systemic diseases.

Taurine is a sulfur-containing amino acid and is maintained in high concentrations in many tissues, as high as submillimolar [6,7]. Taurine is important mainly as an organic osmolyte in mammalian cells; its intracellular concentration is altered by extracellular osmotic pressure, contributing to the maintenance of cell volume [8,9]. In addition, taurine has wide variety of actions in cells, such as antioxidant function, ion dynamics regulation, and the stability of macromolecules, conjugation to bile acid, etc [[10], [11], [12]]. The maintenance of taurine in body is mainly due to its biosynthesis from sulfur-containing amino acids, such as methionine and cysteine, mostly in the liver [13]. Another source of taurine is from dietary intake; especially taurine is rich in seafood [14]. Taurine supplementation benefits many types of pathology, including acute and chronic liver diseases [15]. Taurine ameliorates liver damage induced by alcohol, high fat diet, diabetes, and hepatotoxic medicine [[16], [17], [18], [19]]. Most recently, treatment with taurine has been reported extend life span and health span in mice and health span in monkeys, including improvement of glucose homeostasis and serum liver injury markers [20]. Importantly, taurine treatment attenuates an age-associated increase in senescent markers in several tissues of mice, including liver. Therefore, it is possible that pathological stress-induced cellular senescence may also be attenuated by taurine treatment, which may contribute to the mechanism in preventing disease, but this has not been elucidated.

Carbon tetrachloride (CCl4) is toxic to the liver and is commonly used in experimental models of acute and chronic liver injury [21]. Hepatic taurine content is reduced in chronic CCl4 treatment in mice [22,23]. Recovery of taurine content by taurine administration also acts against CCl4 toxicity by inhibiting liver damage and fibrosis [22,24,25]. In the present study, we examined the effects of taurine on cellular senescence in a chronic model of CCl4-induced hepatotoxicity. We also explored the mechanisms of the antioxidant stress activity of taurine in relation to liver injury and cellular senescence and examined the involvement of H_2_S. In addition, we examined the mechanisms underlying the inhibitory effects of taurine on liver senescence, based on the findings from our previous transcriptome analysis of taurine-treated livers, and discovered an involvement of insulin-like growth factor binding protein-1 (IGFBP-1), a liver specific secretion factor, on the taurine's beneficial role.

## 2 Results

### 2.1 Taurine ameliorates hepatic cellular senescence in CCl4-induced hepatic injury model

**Fig. 1:**
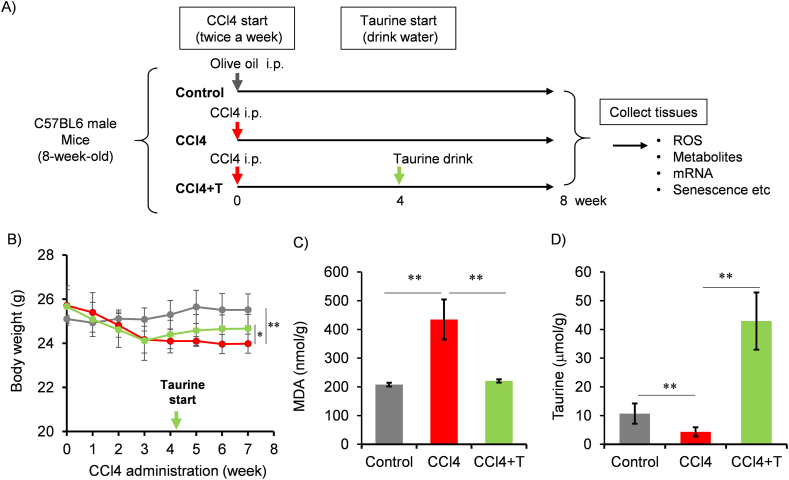
Effect of Taurine on the toxicity induced by chronic CCl4 injection. (A) Flowchart of treatment group. (B)The changes in body weight after CCl4 injection with taurine drinking. Body weight was monitored after CCl4 injection. Taurine treatment by drinking water was started from 4th week of CCl4 administration indicated by arrow. n = 4. (C,D) Hepatic MDA level (C) and taurine concentration (D) were measured. Values shown represent means ± SD. n = 4. ∗∗*p* < 0.01 (post-hoc Bonferroni test). Similar results were observed in another independent experiment as shown in supplemental material.

We investigated the effect of taurine administration on cellular senescence in a CCl4-induced liver injury model. CCl4 was continuously administered for 4 weeks, followed by taurine administration in drinking water and continuous CCl4 administration for additional 4 weeks. The effects on liver injury were analyzed. While body weight of mice was continuously decreased during CCl4 injection, starting taurine administration recovered body weight (Fig. 1A). Previous studies demonstrated that oral taurine treatment reduced oxidative stress markers, including malondialdehyde (MDA), 8-Hydroxy-2′-deoxyguanosine, reduced glutathione, indicating anti-oxidative role of taurine in liver [25,26]. Consistent with these previous reports, MDA level in liver isolated from CCl4-treated mice was higher than control mice, but taurine decreased MDA level (Fig. 1C). Hepatic taurine levels were decreased by 38 % in CCl4-injected mice, but it was recovered by taurine (Fig. 1D). Later, we confirmed that increases in serum aspartate aminotransferase (AST) and alanine aminotransferase (ALT) were significantly suppressed by taurine in CCl4 model (Fig. 5C and D). Therefore, we confirmed that taurine prevents liver toxicity induced by chronic CCl4 injection.

**Fig. 2:**
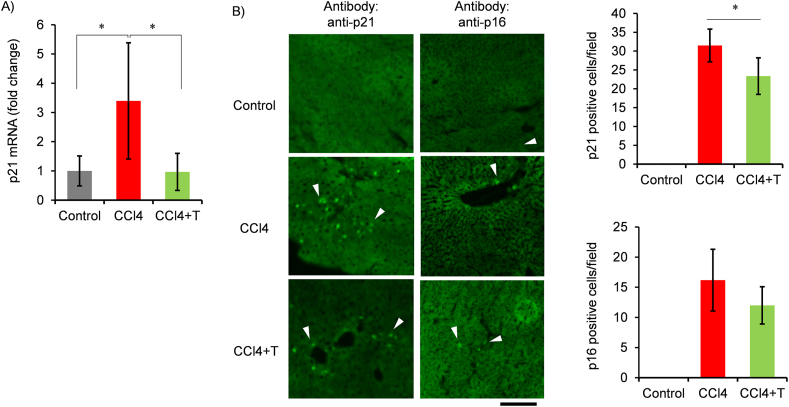
Effect of taurine on the cellular senescence induced by chronic CCl4 injection. (A) p21 expression was measured in the liver by qPCR. The expression level was normalized by the expression level of 28S ribosomal RNA. Values are shown fold of Control. n = 6–8. ∗*p* < 0.05 (post-hoc Bonferroni test). (B) Frozen section of livers was immunostained with anti-p21and anti-p16 antibodies. Arrowheads indicate positive cells. Positive cells per microscopic images were calculated from 5 images per samples for 4 samples for each group. ∗*p* < 0.05 (Student's t-test).

Next, senescent cells in liver tissue were examined. First, senescence markers were detected using RNA extracted from the liver (Fig. 2A). An increase in the cyclin kinase inhibitor p21 was observed in CCl4 group. Taurine administration showed a decrease in p21 expression. P16 was also measured, but we failed to detect an increase in CCl4 (Data not shown). Subsequently, senescent cells in liver tissue section was detected by immunostaining method. While the number of p21-positive cells was increased by CCl4, a decrease in p21-positive cells was observed with taurine administration (Fig. 2B). P16-positive cells were lesser than p21-positive cells in CCl4-injected mice. Numbers of p16 positive cells were not influenced by taurine treatment.

### 2.2 Taurine enhances H_2_S production in liver

**Fig. 3:**
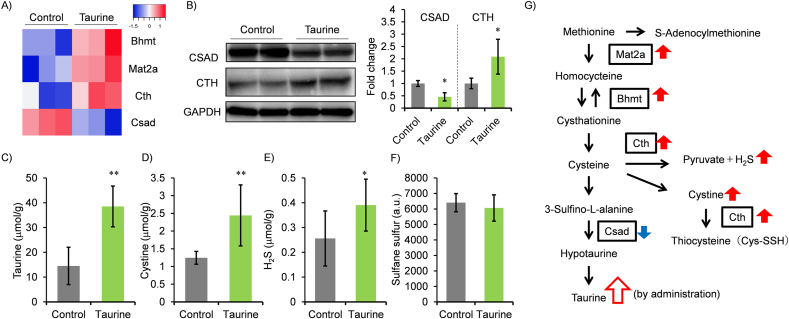
Effect of taurine on cysteine metabolism in liver. (A) Heat maps showing the differentially expressed genes (p < 0.05) which are related to cysteine metabolism in the liver between taurine-treated and control mice, as assessed by microarray previously performed. (B) CSAD and CTH were analyzed by Western blot and band intensity was calculated. The intensity was normalized by the GAPDH level. n = 4. ∗*p* < 0.05 (student's t-test). (C–F) Hepatic taurine, cystine, H_2_S and sulfane sulfur were measured. Values were normalized by tissue weight (g). n = 8 (taurine, cysteine, H_2_S), 4 (sulfane sulfur). ∗*p* < 0.05 (student's t-test). (G) Pathway of cysteine metabolism was shown. Red (increase) and blue (decrease) arrows indicate the effect.

One question that arise from our results is how taurine treatment lowers oxidative liver damage. The sulfur atom of taurine has an oxidation state of +4, and have no ability to scavenge reactive oxygen species (ROS) [27]. Therefore, the antioxidant action of taurine is thought to be due to its ability to inhibit the production of oxidative stress and/or to increase the production of antioxidant metabolites, such as hydrogen sulfide (H_2_S) and glutathione (GSH). It has been reported that increased H_2_S production has also been reported by regulating the expression of H_2_S synthase in the kidney and blood vessels [28,29]. To examine the possibility that H_2_S is involved in anti-oxidative activity of taurine in liver, we first re-analyzed transcriptome data of taurine-treated normal mice which we have already reported [19]. From this transcriptome data, we found an induction of cysteine and methionine metabolism-related genes, such as cystathionine γ-lyase (Cth), methionine adenosyltransferase 2A (Mat2a), and betaine-homocysteine S-methyltransferase (Bhmt) accompanied with a reduction in cysteine sulfinic acid decarboxylase (Csad) (Fig. 3A). Based on these results, it is hypothesized that taurine biosynthesis in liver is eliminated by taurine administration through downregulation of CSAD which is responsible for taurine production from cysteine, and then the production of the other metabolites produced from cysteine, such as H_2_S and thiocysteine (Cys-SSH) which are produced by CTH, is enhanced (Fig. 3G). To examine this, normal mice were treated by 3 % taurine-containing tap water for 4 weeks, and then proteins and metabolites associated with cysteine and methionine metabolism were measured (Fig. 3B–F). Taurine administration increased protein level of cystathionine γ-lyase (CTH) in liver accompanied with a decrease in CSAD (Fig. 3B). Hepatic taurine and cystine are increased by taurine administration (Fig. 3C and D). Moreover, as expected, H_2_S in liver is higher in taurine-treated mice (Fig. 3E). Additionally, we also analyzed sulfane sulfur since an increase in super sulfide species, including Cys-SSH, but its level is not influenced by taurine treatment (Fig. 3F).

**Fig. 4:**
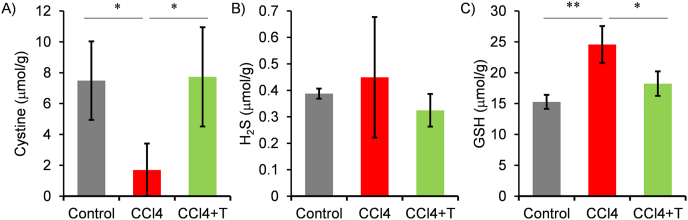
Effect of taurine on cysteine metabolism changes induced by CCl4 injection in liver. (A–C) Hepatic cystine, H_2_S and GSH were measured. Values were normalized by tissue weight (g). n = 4 (cystine), 4 (H_2_S, GSH). ∗*p* < 0.05, ∗∗*p* < 0.01 (post-hoc Bonferroni test). Similar results were observed for cysteine measurement in another independent experiment as shown in supplemental material.

We further examined the effect of taurine on hepatic cystine and H_2_S content in CCl4-treated mice (Fig. 4). While cystine content is reduced in CCl4-treated mice, taurine administration recovered cystine content (Fig. 4A). Since cysteine may be oxidized to produce cystine during sample preparation to measure amino acids, cysteine could not be detected. Meanwhile, inconsistent with the normal mice experiments, an increase in H_2_S by taurine treatment was not confirmed in the CCl4-treated mice (Fig. 4B). A possible reason for this inconsistent results could be that the excess ROS produced by CCl4 eliminated the taurine treatment-induced H_2_S. In addition, we also measured hepatic total GSH content, since a production of antioxidant synthesized from cysteine was expected to increase in CCl4-treated mice. Contrary to expectations, an increase in GSH was observed in CCl4-treated mice, but this was suppressed by administering taurine (Fig. 4C).

**Fig. 5:**
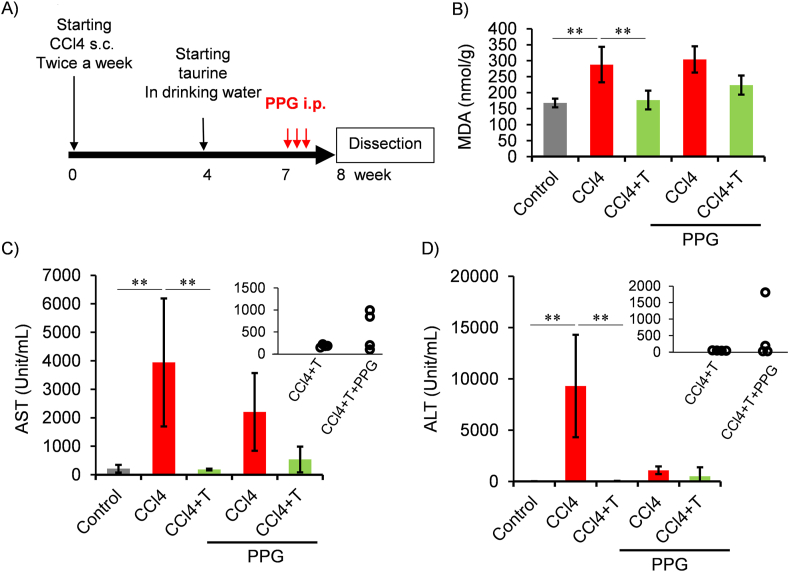
Effect of taurine and PPG on the liver damage induced by CCl4 injection. (A) Time course of CCl4, taurine and PPG injection. (B) Hepatic MDA level were measured. Values shown represent means ± SD. n = 4. ∗*p* < 0.05, ∗∗*p* < 0.01 (post-hoc Tukey-HSD test). (C–D) Serum ALT and AST level were measured. n = 4. ∗*p* < 0.05, ∗∗*p* < 0.01 (post-hoc Tukey-HSD test). Additionally, data from CCl4+taurine (CCl4+T) and CCl4+taurine + PPG (CCl4+T + P)-treated group were picked up into the accompanied scattered graph.

Considering the reduction of cysteine and taurine by CCl4 treatment in the liver, it is assumed that the production of cysteine-associated antioxidants, such as H_2_S and GSH, was enhanced to resist the increased oxidative stress caused by CCl4, with compensatory reductions in cystine and taurine levels. On the other hand, the suppression of taurine synthesis by taurine administration and its antioxidant activity may have reduced cystine consumption.

Additionally, to examine the role of H_2_S on the MDA-lowering effect of taurine, we investigated whether the antioxidant effect of taurine is suppressed by inhibiting H_2_S biosynthesis with CTH inhibitor, propagylglycine (PPG) [30]. PPG was administered only three times in the last week before dissection (Fig. 5A). As a result, PPG treatment tended to attenuate the effect of taurine on lowering MDA which is elevated by CCl4 injection (Fig. 5B). Moreover, while treatment of taurine alone completely prevented inductions of AST and ALT, PPG treatment partially attenuated these prevention (Fig. 5C and D). In detail, 2 of 4 mice treated with both PPG and taurine did not show decrease of AST and ALT, whereas taurine alone completely decreased these biochemical parameters, suggesting the role of CTH on the taurine's hepatoprotective function.

### 2.3 Taurine blocks hepatokine expression in CCl4-induced liver injury

**Fig. 6:**
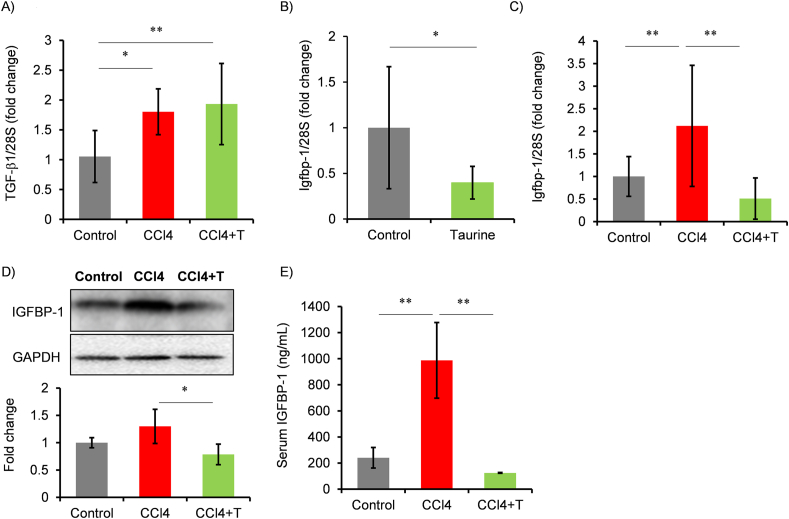
Effect of taurine on TGF−β1 and IGFBP-1 expression in liver. (A) mRNA of TGF−β1 was measured by qPCR. The expression level was normalized by the expression level of 28S ribosomal RNA. Values are shown fold of Control. n = 6–8. (B) Igfbp-1 mRNA expression was measured by qPCR. The expression level was normalized by the expression level of 28S ribosomal RNA. Values are shown fold of Control. ∗*p* < 0.05 (Student's t-test). (C–E) The effect of CCl4 and taurine on Igfbp-1 mRNA (C), protein (D) in liver and serum IGFBP-1 protein (E) were analyzed. n = 4–8. ∗*p* < 0.05, ∗∗*p* < 0.01 (post-hoc Tukey-HSD test).

We investigated whether taurine influence to secretion phenotype (Fig. 6). Senescent-associated secretary phenotype (SASP) influences on the surrounding cells, such as inducing inflammation, additional senescence, and fibrosis, in liver [[2], [3], [4]]. Considering that taurine administration may cause changes in SASP-related factors, we examined SASP in the CCl4 model. While TGF-β1 is induced in CCl4 group, taurine did not attenuate it (Fig. 6A). Meanwhile, induction of IL-1α, IL-1β, IL-6, IL-10, TNF-α were not observed in RNA extracted from the liver of mice treated with CCl4 (Fig. S2).

Next, we explored other secreted factors using transcriptome data used earlier [19]. Re-analysis of this dataset revealed that taurine downregulates IGFBP-1 gene, which is a liver specific protein [31], in normal mice. Quantitative RT-PCR analysis confirmed that taurine administration reduced IGFBP-1 mRNA expression in normal mice (Fig. 6B). Furthermore, in the liver injury model, IGFBP-1 mRNA was elevated after CCl4 administration but suppressed by taurine treatment (Fig. 6C). Protein level of IGFBP-1 in liver tissue was similar between control and CCl4-treated group, while it was lowered by taurine treatment (Fig. 6D). However, importantly, measurement of serum IGFBP-1 levels showed an increase in the CCl4 model, which was also blocked by taurine (Fig. 6E).

## 3 Discussion

**Fig. 7:**
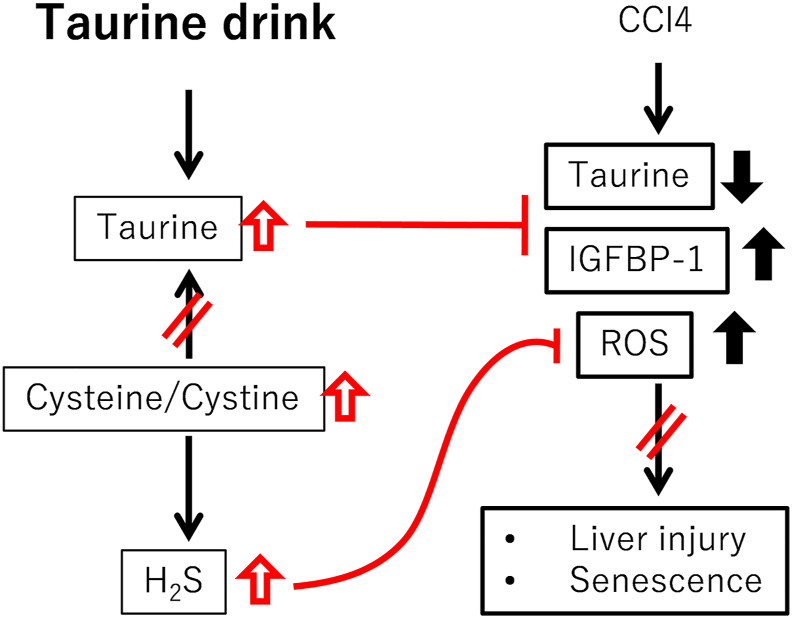
Potential mechanisms underlying anti-senescent role of taurine.

Taurine is well known for its hepatoprotective effects and its role in a variety of liver diseases, whereas cellular senescence has been implicated in liver pathology across these conditions. Since taurine treatment was reported to reduce tissue and cellular senescence accompanied with extending health span and life span [20], we hypothesized that taurine might regulate cellular senescence in liver disease and contribute to disease prevention. Our findings demonstrate that taurine administration reduces the appearance of senescent cells in a CCl4-induced chronic liver injury model. This reduction in cellular senescence was accompanied by attenuation of liver injury, suggesting that taurine mitigates liver injury via senescence suppression. Potential mechanisms underlying anti-senescent role of taurine is summarized in Fig. 7. Importantly, we and the others observed that liver taurine content was decreased by chronic CCl4 injection in mice [22]. Tissue taurine deficiency can accelerate cellular senescence in many tissues, including liver, as evidenced by studies in taurine transporter knockout mice [20,32]. Taken together, stress-induced taurine loss may cause cellular senescence in liver.

Cellular senescence can be triggered by multiple factors, including oxidative stress and DNA damage. Since taurine administration suppressed lipid peroxidation in the CCl4 model, oxidative stress reduction might be one mechanism underlying the decrease in senescent cells. Although taurine itself lacks direct antioxidant activity, it may enhance other antioxidant systems or inhibit oxidative stress-generating pathways, thereby reducing oxidative damage accumulation in the liver. Our study identified a novel role of taurine in increasing hepatic H_2_S levels. This may be due to an increase in cysteine by reducing taurine synthesis from it in liver, as evidenced by a reduction in CSAD expression. Consistent with this suggestion, loss of cysteine dioxygenase, which is one of the main enzyme of taurine biosynthesis pathway, results in an increase in H_2_S concomitant with an impairment of taurine synthesis [33]. H_2_S is known to neutralize ROS and has been reported to suppress cellular senescence [34,35]. Conversely, loss of H_2_S by knocking out CTH in cells causes cellular senescence associated with an increase in p21 [24]. Interestingly, benefit of dietary restriction on health span and life span is mediated by enhanced H_2_S production [36,37]. Therefore, long-term upregulation of H_2_S by taurine may contribute to its anti-senescent effects.

While the effect of taurine in an induction of H_2_S has been previously observed in the kidneys and vasculature [28,29], this is the first report demonstrating it in the liver. However, in the CCl4 model, taurine did not increase H_2_S levels, likely because H_2_S was rapidly consumed to counteract oxidative stress induced in CCl4-treated mice. Pharmacological inhibition of H_2_S synthesis by PPG tended to attenuate the lipid peroxidation-lowering effects of taurine, suggesting that H_2_S contributes to its antioxidant properties. One limitation of our study is that short-term treatment of PPG provided partial evidence for the role of H_2_S in antioxidant function of taurine. Since PPG has undesirable effects, such as reductions in taurine and glutathione and induction in homocysteine [[38], [39], [40]], we did not examine the long term treatment of PPG in taurine-treatment group in the present study. This may be the reason why the effects of PPG on attenuating the beneficial effects of taurine was partial and not significant as shown in Fig. 5. Therefore, further studies with specific, strong and long term inhibition of CTH, such as using CTH knockout mouse, are needed to be clarify the role of H_2_S in anti-senescent effect of taurine in liver.

In this study, we chose HSip-1 fluorescent probe to measure H_2_S in liver. Previous studies used fluorescent probes, including HSip-1, to measure H_2_S in biological samples, such as mouse tissue, blood and cultured cells [[41], [42], [43], [44]]. Sasaoka et al. reported that HSip-1 shows high selectivity and high sensitivity for H_2_S detection; this probe can detect 10 μM H_2_S [45]. Jurkowska et al. has reported that HSip-1 fluorescence after reaction with sodium sulfide is stable for 24 and 48 h, and their experiments by using CDO-null mice and CTH inhibitor supports the validity of this method to measure the production of H_2_S [42]. However, due to the labile nature of H_2_S, the intracellular level of H_2_S remains controversial [45,46]. To calculate precise H_2_S concentrations in liver, it will be necessary to compare various methods in the future work.

Next, we expected CCl4-induced liver injury to promote SASP-related cytokine production. We found an induction of TGF-β in CCl4-treated animals, but taurine did not attenuate it. TGF-β is a prominent SASP factor in chronic liver injury, and chronic TGF-β signaling is associated with the induction of fibrosis [3]. It has also been reported that inhibition of TGF-β suppresses cellular senescence [47], suggesting that TGF-β promotes surrounding cellular senescence. Additionally, TGF-β has been shown to inhibit cancer, indicating a role for increased TGF-β expression by senescent cells in suppressing carcinogenesis [2,48].

Meanwhile, our findings indicate that taurine regulates IGFBP-1 expression. IGFBP-1 is a liver-specific secretory protein that modulates IGF-1 bioavailability [31,49]. It has been reported that IGFBP-1 is induced in conditions such as alcoholic and non-alcoholic liver diseases [50,51]. IGFBP-1 knockout mice exhibited more severe liver injury, including CCl4-induced injury model, accompanied by an increase in hepatic cell death [52], indicating that IGFBP-1 is necessary for liver survival and regeneration. Therefore, the increase in IGFBP-1 caused by CCl4 treatment may be a survival adaptation against lethal stress. On the other hand, the involvement of IGF-1 and IGF system components, including binding proteins, in cellular senescence has been much discussed. Although IGF-1 has been reported to enhance premature senescence, IGF-1 itself may not be a SASP protein [53]. Importantly, the IGF regulatory proteins may function as SASP factor by modulating IGF-1 activity and IGF signaling. IGFBP-1 expression is correlated with increased senescent cell burden in non-alcoholic liver disease model [42]. Since the suppression of IGFBP-1 expression by taurine may be linked to its ability to reduce senescent cells, future studies are required to clarify whether IGFBP-1 promotes liver cell senescence and/or exacerbates liver disease.

Furthermore, circulating IGFBP-1 has been shown to influence other organs. IGFBP-1 has been implicated in hepatic insulin resistance, and lower serum IGFBP-1 levels have been associated with improved glucose tolerance in human clinical studies [54]. Additionally, IGFBP-1 is linked to osteoporosis, cognitive function, muscle mass and all-cause mortality [[54], [55], [56]], suggesting its involvement in systemic age-related disorders. Therefore, taurine may exert broader protective effects by modulating IGFBP-1-mediated liver-to-multi-organ communication, thereby mitigating aging-related functional decline.

The other limitation of our study is that we could not provide conclusive evidence regarding the effect of taurine against fibrosis. Senescence of hepatocytes is associated with the progression of fibrosis and it has been reported that liver fibrosis is suppressed by taurine administration [25], assuming that the reduction of senescent cells by taurine treatment contributes to the suppression of fibrosis. In the present study, we also examined liver fibrosis in CCl4-treated mice (Fig. S3). Although inductions in fibrosis related genes, such as type-1 and type-3 collagen (Col1a1 and Col3a1) and TGF-β, was detected, taurine did not prevent them. The discrepancy between the previous studies and our present results regarding anti-fibrotic role may depend on experiment design, including the dose and duration of CCl4 and/or taurine administration. The study of taurine transporter knockout mice revealed that long term taurine deficiency leads liver fibrosis [53], suggesting that taurine deficiency caused by CCl4 may be associated with fibrosis progression. Detailed studies are needed to elucidate the association between the anti-ageing effect of taurine and liver fibrosis.

The present study has shown that taurine inhibits pathological stress-induced cellular senescence in liver. It may therefore be useful in many liver diseases which the senescent cells contribute to the exacerbation of the disease. Taurine powder is currently used as a prescription drug in Japan to treat liver disease as well as heart failure and mitochondrial diseases. Prescription doses of taurine range from 3 to 12 g per day and have been found to be safe to administer in this range [57,58]. In the 3 % taurine solution used in this study, if mice are to drink 3 mL of water per day, they would have received 4 g of taurine per kg of body weight. This is therefore very much higher than the human dose. However, 3 g has been shown to have a positive effect on the liver in human clinical trials and is considered to be a sufficient dose [59]. In the future, the concentration dependence of taurine, especially dose in human, in the anti-senescence effect via taurine-H_2_S/IGFBP-1 axis should be investigated. Moreover, the temporal relationship needs to be investigated with longer-term experiments to assess the role of taurine treatment in the involvement of H_2_S and IGFBP-1 in chronic liver diseases and systemic age-related disorders.

## 4 Methods

### 4.1 Animal experiments

**Fig. S1:**
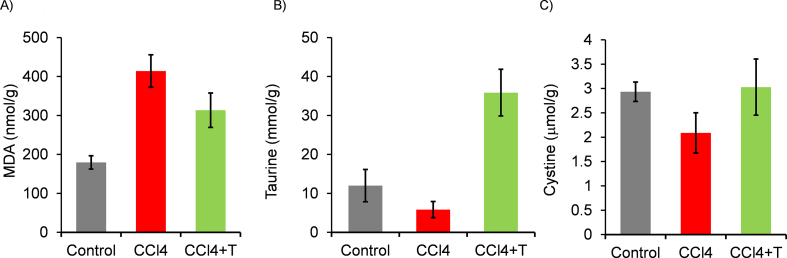
Effect of Taurine on the toxicity induced by chronic CCl4 injection in second CCl4 experiment. (A) Hepatic MDA level (B) taurine and (C) cysteine concentration. Values shown represent means ± SD. n = 2–4.

**Fig. S2:**
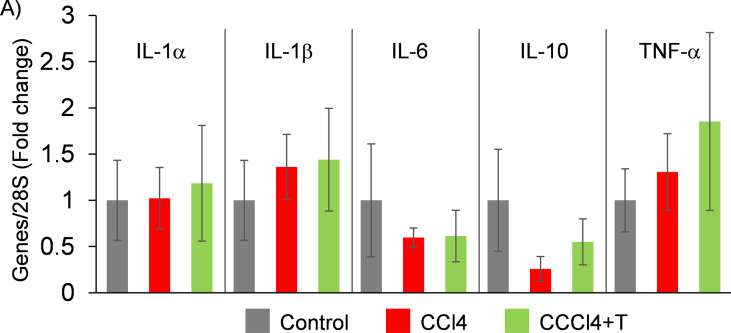
Effect of taurine on SASP factor expression induced by chronic CCl4 injection. Cytokine expressions were measured in the liver by qPCR. The expression level was normalized by the expression level of 28S ribosomal RNA. Values are shown fold of Control. n = 4.

**Fig. S3:**
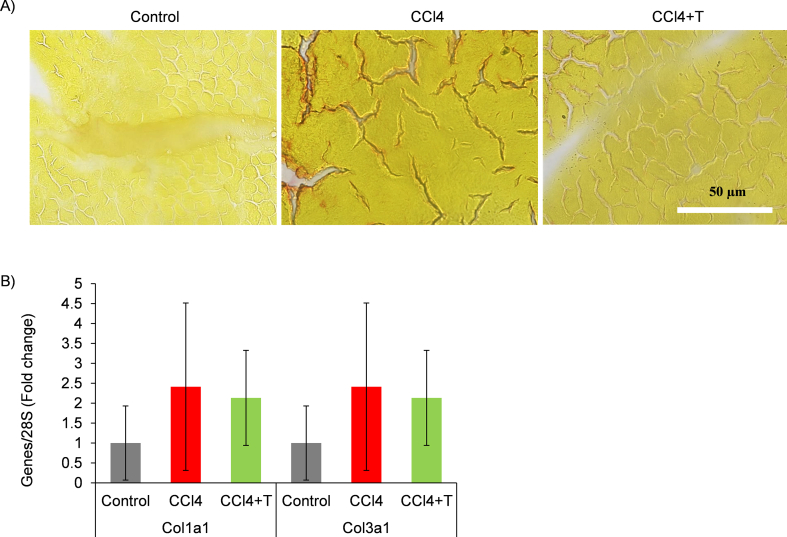
Effect of taurine on fibrosis induced by chronic CCl4 injection. (A) Representative image from Picrosirius red stain. (B) Collagen gene expressions were measured in the liver by qPCR. The expression level was normalized by the expression level of 28S ribosomal RNA. Values are shown fold of Control. n = 4.

All experimental procedures were approved by the Institutional Animal Care and Use Committee of the Fukui Prefectural University. 8-week-old male C57BL/6J mice (Japan Crea, Japan) were used for this study. In CCl4-treatment experiments, 12 animals were divided into three groups, as follows: The control group, the CCl4 group and the CCl4+Taurine group. Mice were treated with intraperitoneal injection of CCl4 (in 20 % olive oil, 2 mL/kg body weight) twice per week for 8 weeks (Fig. 1A). Since some previous studies concerning CCl4-induced chronic liver injury have set the time period at 5–8 weeks [23,25,26], we set it at 8 weeks, the longer period to mainly focus on tissue aging in chronic disease. The control mice were treated intraperitoneally with the same volume of olive oil. Taurine treated animals were received 3 % taurine solution by the drinking water from the 4th week of the CCl4 treatment. The animals of all the recruited groups were weighted before and once in a week and food intake was measured in all treated group 3 times in a week following CCl4-induced liver injury. The same set of CCl4-treatment experiments were repeated twice. Since the data from the two sets of experimental groups could not be considered statistically identical, they were not combined but presented as Supplement data (Fig. S1).

In case of propagylglycine (PPG, Sigma-Aldrich) treatment experiments, animals were divided into five groups, as follows: control group, CCl4 group, CCl4+Taurine group, CCl4+PPG group, CCl4+Taurine + PPG group. PPG (50 mg/g body weight) was intraperitoneally injected 3 times in 8th week as shown in Fig. 5A.

In normal mouse experiments, 16 animals were divided into two groups as follows; control group and taurine group. For taurine group, taurine was administered from tap water containing 3 % taurine. Taurine was kindly provided by Mitsui Chemical Co. Ltd. (Japan). We set 8-week endpoint for taurine intervention to compare control-taurine treatment experiment with CCl4-taurine treatment experiments at the same age of mice.

### 4.2 Biochemical analysis

At the time of sacrifice, blood samples were collected from the orbital sinus under anesthesia with a mixture of medetomidine (0.3 mg/kg bodyweight, Nihon Zenyaku, Japan), midazolam (4 mg/kg body weight, Asteras Phaermaceuticals, Japan), and butorphanol (5 mg/kg body weight, Meiji Pharma, Japan) and immediately centrifuged for 10 min. Liver function tests were performed by using FUJIFILM DRI-CHEM NX700V on serum samples to evaluate the levels of AST and ALT.

### 4.3 MDA measurement

The level of MDA, a measure of lipid peroxidation, was measured by the thiobarbituric acid (TBA) reaction method as described in previous report [60]. 1 mL of 10 % TCA (SIGMA-ALDRICH) and 500 ppm of BHT (Nacalai tesque) were added to 50 mg frozen liver sample. The sample was divided into two portions. One portion was treated with TBA reagent and the other was untreated (without TBA). TBA (0.02 mol/l) (Nacalai tesque) were added to the treated sample. Both samples were heated for 1 h at 95 °C at heat blocker. After cooling add 500 μl Butanol (Fujifilm wako pure chemical corporation) and centrifuge at 15000 rpm for 10min.The absorbance at 535 nm was measured. The absorbance from the untreated sample was subtracted from the TBA-treated sample to calculate the net MDA concentration.

### 4.4 Measurement of taurine, amino acids and total GSH by HPLC

Taurine and amino acids was measured as previously described [61]. In brief, tissue samples were mixed with 10 times volume of 5 % sulphosalytylic acid (SSA) containing the internal standard chemical alpha-aminobutyric acid (0.1 mM, Tokyo Chemical Industry) and were homogenized using a polytron homogenizer. Samples were centrifuged, and the supernatant was neutralized with Na_2_CO_3_ and taurine and amino acids were reacted with phenylisothiocyanate. After dilution with HPLC mobile phase buffer A (10 mM potassium phosphate, pH7.3), samples were subjected to HPLC analysis performed on UltiMate™ 3000 Rapid Separation Binary System (Thermo Fisher, USA). Samples were injected into ODS column (Cosmosil 5C18-MS-II, 4.6mmI.D. x 250 mm, Nacalai tesque). Mobile phases A (10 mM potassium phosphate pH 7.3) and B (acetonitrile) were used. The column oven temperature was set at 40 °C. The flow rate of the mobile phase was 1 mL/min. The gradient of the mobile phase was increased from 5 % to 40 % of buffer B (from 0.3 min to 20 min), and was kept at 100 % of buffer B for 3 min. PITC-derivatized taurine and amino acids were detected by absorption at 254 nm.

Total GSH was measured as previously described [62,63]. Tissues were homogenized in 10 times volume of PBS containing 50 mM ditiothleitol (DTT). PBS was deoxygenated by passing nitrogen bubble for 5 min before use. After centrifuging, 30 μl of supernatant was mix with 70 μl of 100 mM Tris HCl buffer and 10 μl of 20 mM D-penicillamin (Tokyo Chemical Industry). Then, Samples were incubated with 50 μl of 10 mM monobromobimane (mBB) at room temperature for 30 min in the dark. After incubation, samples were mixed with 50 μl of 200 mM SSA and centrifuged at 10,000×*g* (10min, 4 °C)and supernatant was analyzed by HPLC on Prominence HPLC system (Shimazu, Japan). Samples were injected into ODS column (Cosmosil 5C18-MS-II, 4.6mmI.D. x 250 mm, Nacalai tesque). Mobile phases A (0.25 % phosphoric acid in H_2_O, pH 3.9) and B (0.25 % phosphoric acid in methanol) were used. The column oven temperature was set at 40 °C. The flow rate of the mobile phase was 1.2 mL/min. The gradient of the mobile phase was increased from 21 % to 40 % of buffer B (from 3 min to 30 min), and was kept at 100 % of buffer B for 3 min mBB-derivatized compounds were detected by a fluorescence detector (excitation: 370 nm, emission: 485 nm).

### 4.5 Measurement of H_2_S amount

The amount of H_2_S was measured by fluorescence measurement using HSip-1 (Dojindo) as described previously [41,45]. Frozen tissues were homogenized in 10 times volume of the deoxygenated PBS containing HSip-1 (10 μM). After centrifuged at 15,000×*g* (10 min, 4 °C), supernatant was transferred to a 96 well plate. After incubation in the dark at room temperature for 60 min, fluorescence was measured at λem/λex = 491 nm/516 nm.

### 4.6 Sulfane sulfur measurement

Sulfane sulfur was measured by fluorescence measurement using SSP4 (Dojindo) as previously described [64]. Tissues isolated by dissection of mice immediately was homogenized in 10 times volume of PBS containing 0.5 % Triton X-100, sonicated, and centrifuged at 12000 rpm (10 min, 4 °C). Supernatant was mixed with SSP4 (final concentration; 10 μM), and was incubated at 37 °C for 30 min in 96-well plate. Fluorescence was measured at λem/λex = 495 nm/520 nm.

### 4.7 mRNA measurement

Measurement of mRNA expression was performed by real-time reverse transcript-PCR, as previously described [65]. Total RNA was isolated from tissues by using Sepazol super (Nacalai Tesque, Kyoto, Japan) and cDNA was generated by using Rever Tra Ace (Toyobo, Osaka, Japan). Quantitative RT-PCR analysis was performed by using qTOWER iris series Real-time Thermal Cycler (Analytik jena, Germany) with THUNDERBIRD SYBR qPCR Mix NEXT (Toyobo, Japan). The primers used are listed (Table 1).

**Table 1:** Primers used for qRT-PCR.

Name	Forward (F) or Reverse (R)	Sequence
Cdkn1a (p21)	F	5′-TCCAGACATTCAGAGCCACAGG-3′
	R	5′-ACGGGACCGAAGAGACAACG-3′
28S	F	5′- TTAGTGACGCGCGCATGAATGG-3′
	R	5′-TGTGGTTTCGCTGGATAGTAGGT-3′
GAPDH	F	5′- GCCGGTGCTGAGTATGTCGT-3′
	R	5′- CCCTTTTGGCTCCACCCTT-3′
Col1a	F	5′- CCCTGCCGGAGAAGAAGGAA- 3′
	R	5′- TTCACCGGAAGGACCCTTGGGC-3′
Col3a	F	5′- TACACCTGCTCCTGTGCTTCC- 3′
	R	5′-GAAGGCCAGGGTCACCATTTC- 3′
IGFBP-1	F	5′- AGCACCTTGTTCAGCTCCCA- 3′
	R	5′- CAGGATGGGCTGATCCTCGC- 3′
IL-1α	F	5′-AGCAACGTCAAGCAACGGGA-3′
	R	5′-TGCTGATCTGGGTTGGATGGTC-3′
IL-1β	F	5′-TGCCACCTTTTGACAGTGATGAGA-3′
	R	5′- GGACAGCCCAGGTCAAAGGT-3′
IL-6	F	5′- CACTTCACAAGTCGGAGGCTT-3′
	R	5′- GAATTGCCATTGCACAACTCTTTTC-3′
IL-10	F	5′- GTAGAAGTGATGCCCCAGGC-3′
	R	5′-GGAGAAATCGATGACAGCGCC-3′
TGF-β1	F	5′- AGGGCTACCATGCCAACTTCT-3′
	R	5′- CCGGGTTGTGTTGGTTGTACA-3′
TNF-α	F	5′- CAAAATTCGAGTGACAAGCCTGTA-3′
	R	5′- CACCACTAGTTGGTTGTCTTTGAGA-3′

### 4.8 Immunohistochemical analysis

Section from frozen tissues were cut by cryostat (Leica Microsystems, Wetzlar, Germany) and cut into 10 μm. Fixed tissue Section were washed, blocked with Blocking one (Nacalai Tesque), and incubated with primary antibodies diluted 1:100 in Can Get Signal A (TOYOBO). The following antibodies were used: p21 waf1/Cip (12D1) Rabbit mAb (cell signal, #2947), p16 INK4A (E6N8P) Rabbit mAb (cell signal, #18769), Alexa Fluor®488-conjugated goat anti-rabbit IgG (Thermo Fisher scientific, #1622775). Nuclear staining was performed with DAPI, and fluorescence microscopy (BZ-810, Keyence, Japan) was used for observation.

### 4.9 ELISA

ELISA kits for measuring levels of IGFBP-1 in serum were from R&D system a biotech brand (Mouse IGFBP-1 catalog number DY1588-05). The measurement was performed according to the manufacturer's instructions.

### 4.10 Western blot analysis

Western blot was performed as described previously [66]. Frozen tissue was homogenized in lysis buffer and was sonicated for 30s. After centrifuged, supernatant was mixed with SDS-sample buffer (Nacalai Tesque, Japan) for SDS-PAGE. Heat the sample at heat blocker at 95 °C for 5min. The sample was applied to 5–20 % acrylamide gel (Fujifilm Wako pure chemical industries, Ltd) and electrophoresed. Protein blotting was performed by the semi-dry method. The transferred membrane was then blocked by blocking one regeant (Nacalai Tesque) for 20 min. After blocking, the membrane was washed with PBS-T, and then incubated with primary antibodies, which are listed in Table 2, diluted with Can Get signal solution 1 (Toyobo). The membrane was then washed with PBS-T, immersed in secondary antibody diluted with Can Get Signal Solution 2 (Toyobo) and incubated for 1 h. After the antibody reaction was completed, the membrane was washed with PBS-T and the detection reaction was carried out using Chemilumi reagent (Nacalai Tesque) and membrane was photographed under a Chemilumi imaging system (Fusion SL (MS Equipment Co., Ltd)).

**Table 2:** Antibodies used for Western blot.

Antibody	Source	Identifier
CSAD pAb	Thermo Scientific	# PA5-120240
CTH pAb	Proteintech	#12217-1-AP
IGFBP-1 pAb	Proteintech	#31335-1-AP
Rabbit IgG-HRP	Cell Signaling Technology	#7074T
GAPDH (6C5) mAb	Santa Cruz	# SC-32233
mouse IgG-HRP	Santa Cruz	#sc-2031

## 5 Statistics

Data expressed as means ± standard deviation. Statistical analysis was performed by student's t-test or ANOVA followed by post-hoc test. Differences were considered statistically significant when the calculated *p* value was less than 0.05.

## CRediT authorship contribution statement

**Akihiro Tsuboi:** Writing – original draft, Methodology, Investigation, Formal analysis, Data curation. **Hamida Khanom:** Writing – original draft, Methodology, Formal analysis, Data curation. **Riki Kawabata:** Methodology, Investigation, Data curation. **Takanori Matsui:** Supervision. **Shigeru Murakami:** Validation, Conceptualization. **Takashi Ito:** Writing – review & editing, Writing – original draft, Visualization, Validation, Software, Resources, Project administration, Methodology, Investigation, Funding acquisition, Formal analysis, Data curation, Conceptualization.

## Statement

During the preparation of this work the authors used DeepL in order to translate and improve language. After using this tool, the authors reviewed and edited the content as needed and takes full responsibility for the content of the publication.

## Declaration of competing interest

The authors declare that they have no known competing financial interests or personal relationships that could have appeared to influence the work reported in this paper.

## Figures and Tables

**Fig. S4:**
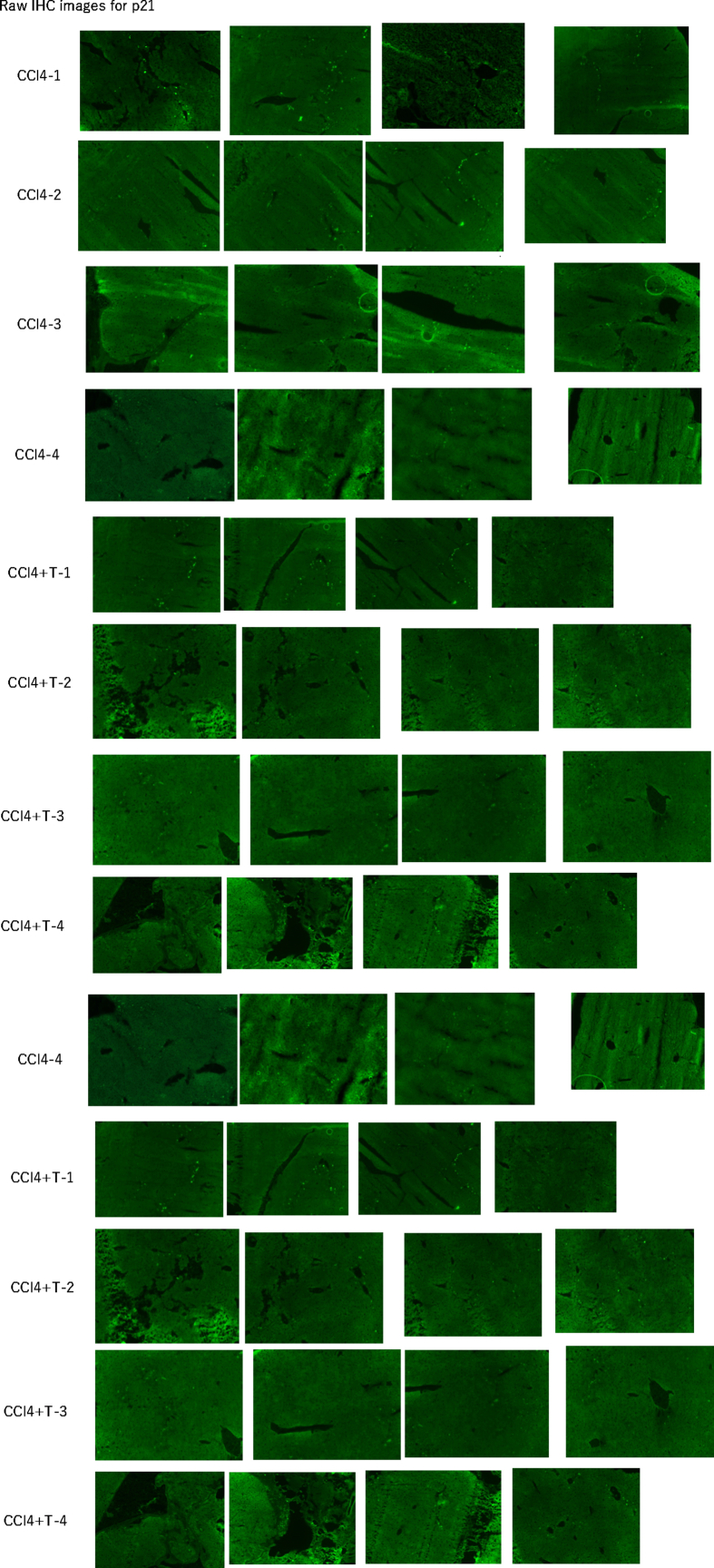
Raw IHC images for p21 in liver sections.

**Fig. S5:**
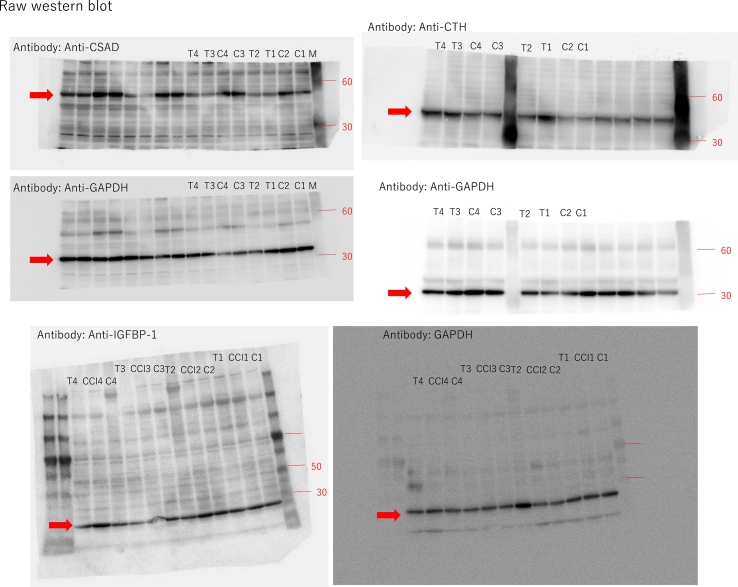
Rraw western blot images.

## Data Availability

Data will be made available on request.
